# Potential diagnostic value of PD‐1 in peripheral blood mononuclear cells of postmenopausal osteoporosis patients

**DOI:** 10.1002/jcla.23223

**Published:** 2020-02-17

**Authors:** Xiu‐Ping Cai, Qing Zhao, Zhao‐Di Guo, Shao‐Jun Lin, Zhi‐Xiang Chen, Min‐Yuan Chen, Lei Zheng, Ke‐Wei Zhao

**Affiliations:** ^1^ The Third Affiliated Hospital of Guangzhou University of Chinese Medicine Guangzhou China; ^2^ The Third Clinical College of Guangzhou University of Chinese Medicine Guangzhou China; ^3^ Guangzhou University of Chinese Medicine Guangzhou China; ^4^ Nanfang Hospital Southern Medical University Guangzhou China

**Keywords:** bone turnover markers, diagnostic value, peripheral blood mononuclear cell, programmed cell death protein 1, postmenopausal osteoporosis

## Abstract

**Background:**

Postmenopausal osteoporosis (PMOP) is an estrogen deficiency‐induced skeletal disorder. Bone mineral density (BMD) testing is the gold standard for diagnosing osteoporosis. However, its sensitivity for fracture risk assessment is low. Programmed cell death protein 1 (PD‐1) is a key immune checkpoint molecule implicated in the pathophysiology of bone remodeling, but its role in osteoporosis has not yet been explored. Thus, this study aimed to assess the expression and diagnostic utility of PD‐1 in PMOP.

**Methods:**

A total of 56 patients with PMOP and 37 postmenopausal healthy controls (NC) were enrolled in the study. Peripheral blood mononuclear cells (PBMCs) were isolated by Ficoll density gradient centrifugation, and PD‐1 expression was measured by quantitative polymerase chain reaction (qPCR). Pearson's correlation test was performed to explore the associations between PD‐1 level and clinical variables, while receiver operating characteristic (ROC) curve analysis was used to evaluate the potential diagnostic value of PD‐1 in patients with PMOP.

**Results:**

We found that PD‐1 level was significantly upregulated in the PBMCs of PMOP patients than those of NC (*P* = .016). PD‐1 expression was positively correlated with C‐reactive protein (CRP) levels. ROC curve analysis showed that PD‐1 had certain diagnostic value for PMOP (area under the curve = 0.65, standard error = 0.06, 95% confidence interval [0.53,0.76], *P* = .016), with a sensitivity and specificity of 44.64% and 81.08%, respectively.

**Conclusion:**

Programmed cell death protein 1 is significantly upregulated in the PBMCs of PMOP patients and has certain diagnostic value for PMOP.

## 1 INTRODUCTION

Postmenopausal osteoporosis (PMOP) is a systemic, metabolic bone disorder resulting from decreased ovarian estrogen production after menopause^1^ and disrupted balance between bone resorption (mediated by osteoclasts) and bone formation (mediated by osteoblasts). Estrogen deficiency can manifest as increased bone resorption^2^ and osteoporotic fractures, which can seriously affect the quality of life of middle‐aged and elderly women. Bone mineral density (BMD) testing using dual‐energy X‐ray absorptiometry (DXA) is the gold standard for diagnosing osteoporosis. However, the technique is limited because changes to bone density occur slowly and can only be measured meaningfully over the course of several years. Measurement of bone turnover markers (BTMs) is useful in differential diagnosis, distinguishing the different types of osteoporosis, and early assessment of patient response to treatment, but it cannot be used to diagnose osteoporosis. Therefore, there is an urgent need to identify more sensitive diagnostic markers for osteoporosis.

The recent advances in osteoimmunology, an interdisciplinary research field that explores the interaction between bone and immune system, revealed that bone remodeling is under strict immunological control.^3^ Thus, it is of vital clinical importance to identify the specific immune molecules involved, as they could serve as potential targets for the diagnosis and treatment of osteoporosis.

Programmed cell death protein‐1 (PD‐1) is an inhibitory immune receptor that is mainly expressed on active T cells, B cells, monocytes, dendritic cells, and natural killer cells. PD‐1 has two ligands, of which programmed death ligand‐1 (PD‐L1) is considered to be the primary ligand. The PD‐1/PD‐L1 pathway plays a critical role in the pathophysiology of autoimmune diseases, tumorigenesis, chronic infections, and inflammation.^4^ A recent study found that activation of the PD‐1/PD‐L1 pathway was associated with hyper‐activation of osteoclasts and impaired T‐cell proliferation in multiple myeloma.^5^ However, its role in osteoporosis has not yet been studied. To the best of our knowledge, no clinical studies to date have investigated PD‐1 levels in peripheral blood mononuclear cells (PBMCs) of osteoporosis patients. Therefore, we explored PD‐1 expression, and its association with clinical and laboratory parameters, in PMOP patients.

## 2 MATERIALS AND METHODS

### 2.1 Ethical approval

A total of 93 postmenopausal women, including 56 patients with PMOP and 37 healthy controls, were enrolled in this study according to the inclusion and exclusion criteria listed below. The subjects were all patients of the Third Affiliated Hospital of Guangzhou University of Chinese Medicine between May 2017 and September 2018. All aspects of the study were approved by The Human Research Ethics Committee of the Third Affiliated Hospital of Guangzhou University of Chinese Medicine, and informed written consent was obtained from all study participants.

### 2.2 Study inclusion criteria

(a) Postmenopausal osteoporosis diagnosed according to 1998 World Health Organization (WHO) criteria; (b) menopause onset within 5‐10 years prior; (c) age below 70 years; (d) bone mineral density (BMD) measured by dual‐energy X‐ray absorptiometry (DXA); and 5) T‐score at the lumbar spine or femur ≤ −2.5 standard deviations (SD).

### 2.3 Study exclusion criteria

(a) Patients with non‐menopausal osteoporosis or other metabolic bone diseases; (b) patients treated in the past six months with estrogen, progesterone, or other drugs that affect bone metabolism; (c) patients with endocrine diseases that can cause secondary osteoporosis; and (d) patients with cancer or severe heart, blood, or mental disorders.

### 2.4 Isolation of PBMCs

A sample of venous blood was collected from all participants. The PBMCs were isolated by density gradient separation at 18‐20°C using Ficoll‐Paque PLUS (GE Healthcare), according to the manufacturer's instructions. The PBMCs were isolated within 6 hours of blood collection to optimize their viability.

### 2.5 Total RNA extraction

Total RNA was extracted from the isolated PBMCs using TRIzol reagent (Invitrogen, Karlsruhe, Germany) according to the manufacturer's instructions.

### 2.6 Reverse transcription

RNA was reverse transcribed into cDNA using PrimeScript RT Master Mix (Perfect Real Time) reverse transcription polymerase chain reaction (RT‐PCR) kit (Takara Bio). The cDNA was diluted with 60 μL of ddH_2_O and stored at −20°C until analysis.

### 2.7 Quantitative PCR (qPCR)

Polymerase chain reaction was performed on the Rotor‐Gene Q qPCR cycler (QIAGEN), using the UltraSYBR Mixture (High ROX) (CWBiotech). The PCR primers were designed and synthesized by Sangon Biotech Co., Ltd. (Table 1). The relative expression of PD‐1 was normalized to that of human β‐actin, using the 2^−ΔΔCT^ method.

**Table 1:** PCR primer sequences for the target and housekeeping genes

	Forward primer	Reverse primer
PD‐1	CCAGGATGGTTCTTAGACTCCC	TTTAGCACGAAGCTCTCCGAT
β‐Actin	TGACGTGGACATCCGCAAAG	CTGGAAGGTGGACAGCGAGG

Abbreviations: PCR, polymerase chain reaction; PD‐1, programmed cell death protein 1.

### 2.8 Statistical analyses

All statistical analyses were performed using GraphPad Prism version 6.0. Continuous variables were expressed as mean ± SD and compared between the groups using unpaired *t* test. Differential PD‐1 expression was analyzed by *t* test, and the results were presented as a scatter plot. Linear associations between parameters were evaluated using Pearson's correlation. The diagnostic value of PD‐1 was assessed by receiver operating characteristic (ROC) curve analysis, in which the PMOP patients and healthy controls served as true positives and true negatives, respectively. The difference was considered statistically significant when *P* < .05.

## 3 RESULTS

### 3.1 Characteristics of study subjects

Participant age, white blood cell count (WBC), C‐reactive protein (CRP), lymphocyte count (LYM), and erythrocyte sedimentation rate (ESR) of the menopausal women did not differ significantly between the PMOP and control groups (Table 2).

**Table 2:** Demographic and clinical characteristics of patients with PMOP and healthy controls

Item	Control (n = 37)	PMOP (n = 56)	*P*‐Value
Age (years)	61.89 ± 5.79	62.33 ± 5.57	.716
WBC (10^9/^L)	7.14 ± 2.13	6.79 ± 2.11	.452
CRP (mg/L)	42.19 ± 27.07	37.17 ± 26.38	.442
LYM (10^9^/L)	1.98 ± 0.60	2.26 ± 0.90	.099
ESR (mm/h)	42.19 ± 27.07	37.77 ± 26.38	.442

Abbreviations: CRP, C‐reactive protein; ESR, erythrocyte sedimentation rate; LYM, lymphocyte count; PMOP, postmenopausal osteoporosis; WBC, white blood cell count.

### 3.2 Expression of bone turnover markers (BTMs)

The T‐score at the lumbar spine or femur, and expression of bone turnover markers (BTMs), differed significantly between the two groups. The levels of procollagen type 1 N‐terminal propeptide (PINP), osteocalcin (OSTEOC), and β‐crosslaps (CROSSL) were higher in patients with PMOP than in controls (Table 3).

**Table 3:** Levels of bone turnover markers in patients with PMOP and healthy controls

	Control (n = 37)	PMOP (n = 56)	*P*‐Value
T‐score	−1.50 ± 0.75	−3.28 ± 0.59	.000
BMD (g/cm^2^）	0.87 ± 0.11	0.67 ± 0.09	.000
PINP (ng/mL)	43.93 ± 18.71	61.88 ± 21.11	.001
CROSSL (ng/mL)	0.66 ± 0.31	0.91 ± 0.44	.019
OSTEOC (ng/mL)	15.75 ± 6.20	20.19 ± 9.36	.028

Abbreviations: BMD, bone mineral density; CROSSL, β‐crosslaps; OSTEOC, osteocalcin; PINP, procollagen type 1 N‐terminal propeptide; PMOP, postmenopausal osteoporosis.

### 3.3 PD‐1 expression

**Figure 1:**
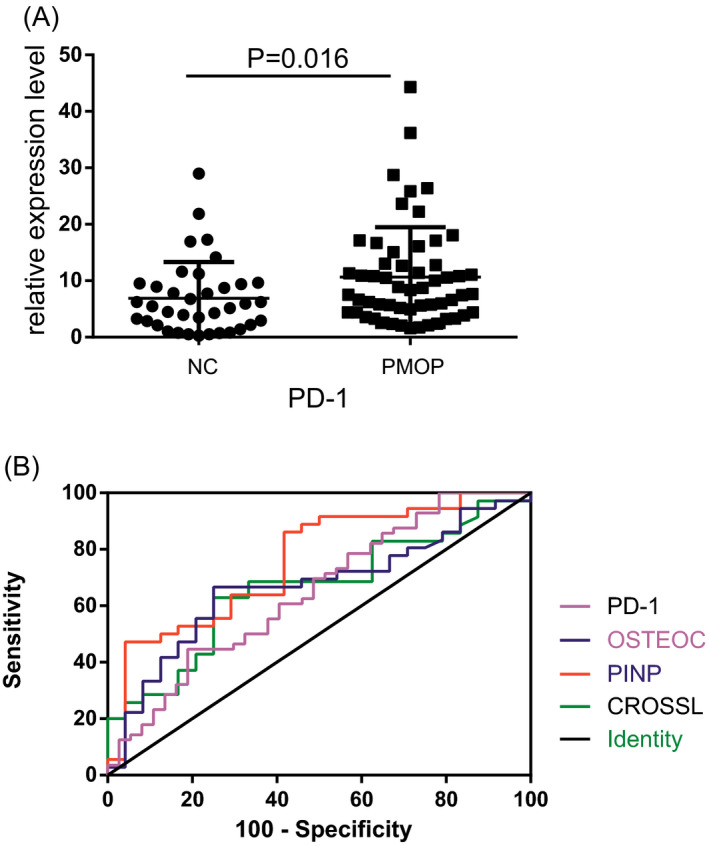
A, PD‐1 expression in the PBMCs of patients with PMOP (n = 56) and healthy postmenopausal women (n = 37), as measured by qPCR. B, Diagnostic value of PD‐1, PINP, OSTEOC, and CROSSL for PMOP, as assessed by ROC curve analysis

We performed qPCR to measure the expression levels of PD‐1 in the PMOP and control groups. PD‐1 was significantly upregulated in the PMOP group compared with the control group (*P* = .016) (Figure 1A).

### 3.4 Correlation between PD‐1 expression and clinical variables

To determine whether PD‐1 in the PBMCs of PMOP patients could serve as a biomarker for the severity of PMOP, we evaluated the correlations between PMOP‐related clinical features and PD‐1 expression in PMOP patients. PD‐1 level was correlated with CRP level (*r* = .334, *P* < .05), but not with patient age, WBC count, LYM, ESR, BMD, T‐score, or CROSSL, PINP, or OSTEOC expression (Table 4).

**Table 4:** Pearson correlation coefficients of the measured variables

	Age	WBC	LYM	ESR	CRP	CROSSL	PINP	OSTEOC	BMD	T‐score	PD‐1
Age		0.223	0.372 **	0.007	−0.133	−0.308	0.095	−0.341*	−0.124	−0.089	−0.026
WBC			0.705**	0.237	0.260	−0.076	−0.149	−0.146	0.010	−0.017	0.015
LYM				0.023	−0.121	−0.150	−0.041	−0.189	0.150	0.247	0.098
ESR					0.353**	0.356*	−0.043	0.298	−0.026	−0.100	0.087
CRP						−0.054	0.006	0.233	−0.285	−0.203	0.334*
CROSSL							0.131	0.396*	0.195	0.028	0.001
PINP								0.418*	0.066	−0.144	0.047
OSTEOC									0.087	0.155	−0.098
BMD										0.666**	−0.236
T‐score											−0.072

Abbreviations: BMD, bone mineral density; CROSSL, β‐crosslaps; CRP, C‐reactive protein; ESR, erythrocyte sedimentation rate; LYM, lymphocyte count; OSTEOC, osteocalcin; PINP, procollagen type 1 N‐terminal propeptide; WBC, white blood cell count.

* *P* < .05

** *P* < .01

### 3.5 ROC curve analysis

We performed ROC curve analysis to assess the diagnostic value of PD‐1 for PMOP. The results showed that the level of PD‐1 in the PBMCs could distinguish PMOP patients from healthy controls (Figure 1B). The area under the ROC curve (AUC) was 0.65 (standard error (SE) = 0.06, 95% confidence interval (CI) [0.53,0.76], *P* < .05), indicating a high specificity (81.08%), but low sensitivity (44.64%) for PMOP. In comparison, the AUC for PINP, OSTEOC, and CROSSL was 0.76, 0.67, and 0.66, respectively (Table 5).

**Table 5:** ROC curve parameters for PD‐1 and BTMs

	AUC	Standard Error	95% CI	*P*‐Value	Youden Index	Sensitivity (%)	Specificity (%)	Cutoff
CROSSL	0.66	0.07	0.52‐0.80	.037	0.38	62.86	75.00	0.78
PINP	0.76	0.06	0.63‐0.88	.001	0.44	86.11	58.33	44.93
OSTEOC	0.67	0.07	0.53‐0.81	.029	0.42	66.67	75.00	16.49
PD‐1	0.65	0.06	0.53‐0.76	.016	0.26	44.64	81.08	9.84

Abbreviations: AUC, area under the ROC curve; BTMs, bone turnover markers; CI, confidence interval; CROSSL, β‐crosslaps; OSTEOC, osteocalcin; PD‐1, programmed cell death protein 1; PINP, procollagen type 1 N‐terminal propeptide.

## 4 DISCUSSION

Postmenopausal osteoporosis is a common bone disease characterized by reduced bone mass, microstructural destruction of bone tissue, increased bone fragility, and high risk of fracture. It usually begins 5‐10 years after the onset of menopause and can seriously affect a woman's quality of life.^6^ BMD measurements by DXA remain the gold standard for the diagnosis of osteoporosis. However, a number of risk factors for developing osteoporosis and fractures are BMD‐independent.^7^ Furthermore, many patients undergoing BMD testing may not be recognized as having a higher fracture risk, because their T‐scores signify osteopenia (reduced bone density without full osteoporosis). Importantly, a T‐score of between −1.0 and −2.5 can indicate an increased risk of fracture if risk factors such as advanced age or prior history of fracture are present.^8^ It follows that sole reliance on BMD testing may delay diagnosis and treatment of osteoporosis. BTMs, such as PINP, OSTEOC, and CROSSL, are used to predict fracture risk and monitor the efficacy of antiresorptive therapy. However, these markers are subject to several sources of variability, including patient's age, gender, pregnancy, circadian rhythm, food intake, and recent fracture. Variable expression, combined with the lack of international reference standards, means that BTMs cannot be used to diagnose osteoporosis, nor do they improve prediction of bone loss or fracture.^9^ The National Bone Health Alliance (NBHA) recommends that sample handling and patient preparation for CROSSL and PINP measurements are standardized to reduce pre‐analytical variability.^10^ Controllable and uncontrollable patient factors also need to be reviewed to facilitate the interpretation of results and minimize pre‐analytical variability.^11^ Michelsen et al^12^ and Vasikaran et al^13^ called for a need for international reference standards to address the uncertainty in the clinical use of BTMs. Our study found that the levels of PINP, OSTEOC, and CROSSL were significantly increased in patients with PMOP than in healthy controls (*P* < .05). Out of the three markers, PINP had the largest AUC, the highest sensitivity, but the lowest specificity for diagnosing PMOP. Although PINP has been proposed as a reference marker for bone formation, PINP assays must first be harmonized to ensure comparability of results between laboratories.^14^

T cells, which are closely related to osteoblasts and osteoclasts, can affect bone metabolism by releasing osteoclastogenic cytokines and factors that stimulate bone formation. Activated T cells play an important role in bone destruction and PMOP.^15^, ^16^ As an important immunosuppressive molecule, PD‐1 is mainly expressed on activated T cells. Raptopoulou et al^17^ found that PD‐1 null mice demonstrated increased incidence and greater severity of collagen‐induced arthritis than wild‐type mice, and this effect was associated with enhanced T‐cell proliferation and increased production of cytokines, such as interferon‐γ (IFN‐γ) and interleukin‐17 (IL‐17). The PD‐1 pathway regulates peripheral T‐cell responses in both human and murine arthritis. Liu et al^18^ reported that in rheumatoid arthritis (RA), inflammatory cytokines, including tumor necrosis factor‐α (TNF‐α) and IFN‐γ, promoted the expression of soluble PD‐1 in the plasma. Inflammatory mediators, such as TNF‐α, IL‐1, IL‐6, IL‐7, and IL‐17, are known to upregulate macrophage colony‐stimulating factor (M‐CSF) and receptor activator of nuclear factor κ‐B ligand (RANKL), stimulate osteoclasts, and inhibit osteoblasts, while their abnormal activation can induce osteoporosis.^3^ RA is a major risk factor for osteoporosis,^19^ such that the incidence of osteoporosis is doubled in patients with RA compared to the general population.^20^ Therefore, we speculate that PD‐1/PD‐L1 signaling is involved in the pathogenesis of osteoporosis by upregulating the expression of inflammatory factors, stimulating osteoclasts, and inhibiting osteoblastic bone formation. In this study, the non‐specific inflammatory indicators (WBC count, CRP level, LYM, and ESR) did not significantly differ between the PMOP group and the control group (*P* > .05). A possible explanation for this finding is that PMOP is not associated with systemic inflammation.

In our study, PD‐1 was upregulated in the PBMCs of PMOP patients compared to those of healthy controls. Nagahama et al^21^ found that PD‐1 deficiency inhibited osteoclast formation, leading to mild osteopetrosis in mice. In another study, Hao Zhang et al^22^ reported that the expression of PD‐1 was increased in aged mice after hip fracture and surgery and that antibody blockade of PD‐1 can partially restore T‐cell function. As we all known, PMOP may lead to fracture. Both of the two animal experiment results suggested that PD‐1 may be involved in the pathogenesis and development of PMOP. In addition, we found that PD‐1 in PBMCs had potential diagnostic value for PMOP (AUC = 0.65, SE = 0.06, 95% CI [0.53,0.76], *P* < .05). Compared to traditional BTMs, PD‐1 in PBMCs had the highest specificity (81.08%), but lowest sensitivity (44.64%) for PMOP diagnosis. In an earlier study, we used enzyme‐linked immunosorbent assays (ELISA) to assess the expression of PD‐1 and PD‐L1 in the serum of patients with PMOP (n = 21) and healthy controls (n = 20). Neither PD‐1 levels (15.37 ± 3.49 vs 27.58 ± 7.93, respectively) or PD‐L1 levels (9.44 ± 2.33 vs 16.79 ± 4.31, respectively) differed significantly between the groups (*P* = .160; *P* = .137, respectively) (own unpublished observations), which suggested that measuring PD‐1 level in the PBMCs might be more clinically useful than measuring it in the serum.

There are several limitations to this study. Firstly, the number of patients was relatively small and derived from a single center. Larger studies in different racial and regional groups are warranted. Secondly, diagnostic utility of PD‐1 to distinguish PMOP from senile osteoporosis, secondary osteoporosis, and other orthopedic diseases was not evaluated. Finally, the role of PD‐1 in PMOP was not explored in vivo. Our future studies will endeavor to address these shortcomings.

To the best of our knowledge, we are the first to determine the expression of PD‐1 in the PBMCs of PMOP patients. Our findings help to explain the role of PD‐1 in the pathogenesis and progression of the disease, and suggest that measurement of PD‐1 levels in the PBMCs has potential diagnostic application for PMOP. Future studies should explore the molecular mechanisms underlying the role of PD‐1 in PMOP.

## AUTHOR CONTRIBUTIONS

X‐P C involved in total RNA extraction, reverse transcription, quantitative PCR, statistical analysis, and paper writing; Q Z performed PBMC isolation and clinical data collection; Z‐D G performed PBMC isolation; S‐J L involved in total RNA extraction and reverse transcription; Z‐X C involved in patient enrollment; M‐Y C carried out BMD measurement; L Z is the project supervisor; K‐W Z is the project leader.
